# Recent advances in bio-based multi-products of agricultural Jerusalem artichoke resources

**DOI:** 10.1186/s13068-018-1152-6

**Published:** 2018-06-01

**Authors:** Yibin Qiu, Peng Lei, Yatao Zhang, Yuanyuan Sha, Yijing Zhan, Zongqi Xu, Sha Li, Hong Xu, Pingkai Ouyang

**Affiliations:** 1 College of Food Science and Light Industry, Nanjing Tech University, Nanjing, 211816 China; 2 Nanjing Institute for Comprehensive Utilization of Wild Plants, Nanjing, 210042 China; 3 Jiangsu National Synergetic Innovation Center for Advanced Materials, Nanjing Tech University, Nanjing, 211816 China

**Keywords:** Jerusalem artichoke, Inulin, Inulinase, Non-grain fermentation, Biorefinery

## Abstract

The Jerusalem artichoke is a perennial plant that belongs to the sunflower family. As a non-grain crop, Jerusalem artichoke possesses a number of desirable characteristics that make it a valuable feedstock for biorefinery, such as inulin content, rapid growth, strong adaptability, and high yields. This review provides a comprehensive introduction to renewable Jerusalem artichoke-based biomass resources and recent advances in bio-based product conversion. Furthermore, we discuss the latest in the development of inulinase-producing microorganisms and enhanced inulin hydrolysis capacity of microbes by genetic engineering, which lead to a more cost-effective Jerusalem artichoke biorefinery. The review is aimed at promoting Jerusalem artichoke industry and new prospects for higher value-added production.

## Background

Concerns over fossil fuels depletion and environmental protection have attracted increasing worldwide interest in the development and utilization of sustainable and taintless energy resources. A sunflower species native to North America, *Helianthus tuberosus* L., also known as the Jerusalem artichoke, is a potential source of renewable energy [1]. Meanwhile, the increasing use of chemical fertilizers and development of irrigation agriculture have led to the constant expansion of secondary salinization land area [2]. Because of more tolerance to harsher conditions than that of most commercial crops, Jerusalem artichoke was selected and developed for large-scale cultivation in saline–alkaline soils or coastal shoals [3, 4]. Therefore, the establishment of this cover crop in non-cultivatable land can reap both ecological and economic benefits.

**Fig. 1:**
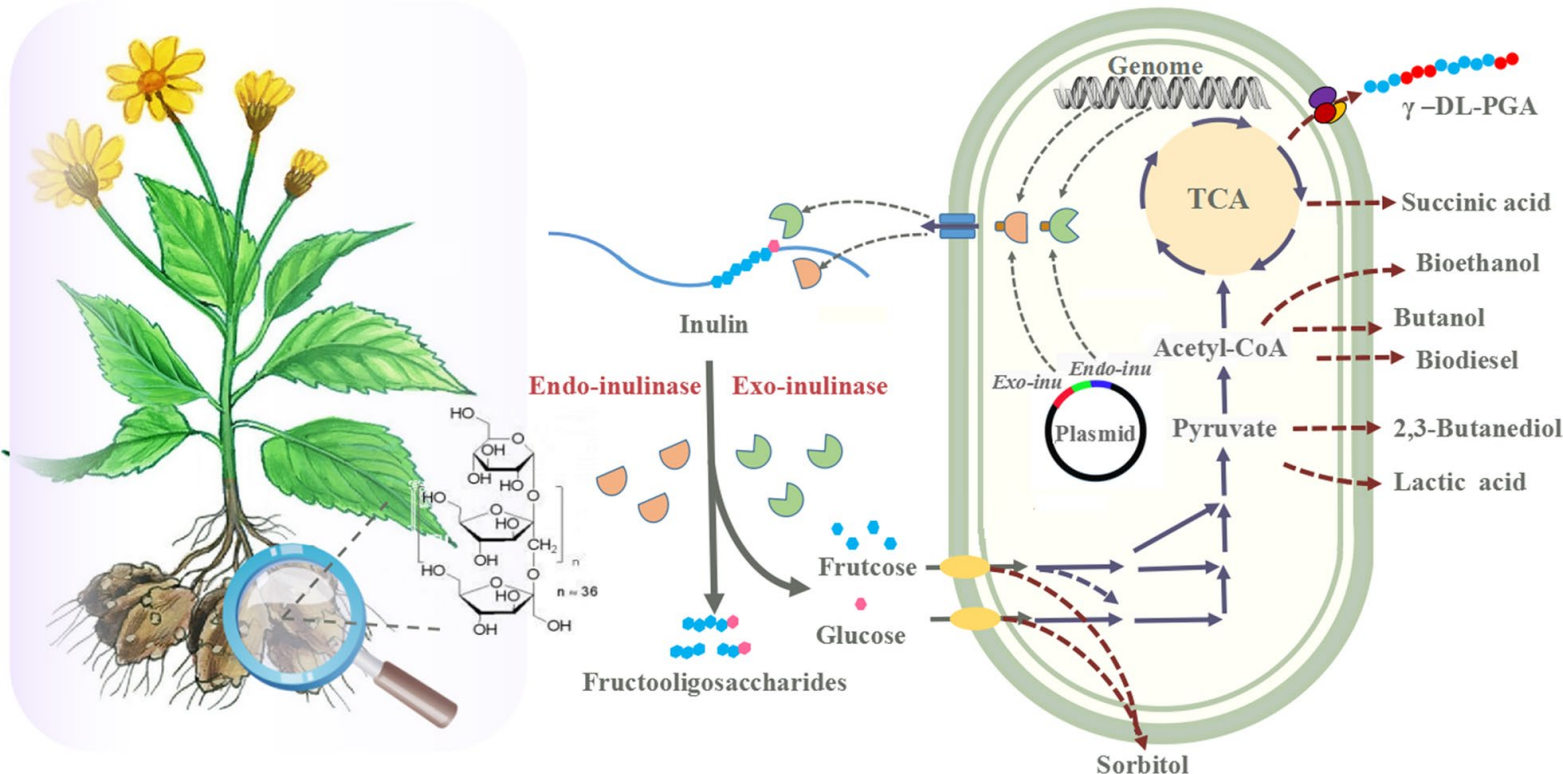
Schematic diagram of the biorefinery of Jerusalem artichoke biomass

In addition to its unique value in ecological environment improvement, inulin-rich Jerusalem artichoke serves as an important raw material in the food, chemical, and pharmaceutical industries (Fig. 1) [5, 6]. A promising candidate, Jerusalem artichoke exhibits many advantages for biorefinery. Compared with traditional grain crops, the Jerusalem artichoke produces large amounts of biomass and can be harvested three times a year [7]. Furthermore, the inulin found in Jerusalem artichoke tubers can be easily processed using available technologies, and can be suitable as a substrate for biorefinery. More importantly, the non-food utilization of this biofuel crop does not compete for arable land with grain crops cultivated for food production [8]. This review aims to provide a comprehensive overview on the characteristics of Jerusalem artichoke and the pretreatment process for biorefinery, as well as a description of inulinase-producing microorganisms and inulinase catalytic mechanisms that will provide fundamental knowledge for inulin biorefinery studies. Furthermore, recent advances in Jerusalem artichoke research and development of bioproducts derived from these artichokes are summarized. Finally, we discuss the prospects of energy crops in future biological manufacturing.

## Overview of the Jerusalem artichoke resource

### Jerusalem artichoke features

Jerusalem artichoke (*Helianthus tuberosus*) is a perennial herbaceous plant that belongs to the Compositae family [9, 10]. Due to its strong adaptability, Jerusalem artichoke is widely distributed in China as a cultivated crop [11]. Morphologically, the Jerusalem artichoke is large, gangly, and highly branched with yellow flower heads. The young stems are stout and can grow 100–300 cm tall. It has opposite leaves on the lower part of the stem that are roughly 10–20 cm long and 5–10 cm in width, and are believed to be important for tuber yields [12]. The Jerusalem artichoke has a hairy and fibrous root system which can grow as long as 127 cm, showing strong environmental tolerance and effective acquisition of soil nutrients [13]. The tubers are irregularly spherical or spindle shaped and vary in color from pale brown to white, red, or purple [14]. As an easily grown crop, it endures a wider range of temperatures and can tolerate pH levels ranging from 4.5 to 8.2 [13]. In addition, cultivation of Jerusalem artichoke does not need too much management and protection due to its strong resistance to pests and plant diseases [15]. It can be harvested annually after planting, leading to high yields of fresh weight (90 t) per hectare that can be used as a sustainable biomass feedstock for biorefinery [16].

### Jerusalem artichoke components

Jerusalem artichoke is a semi-wild plant resource with a great potential for development. Jerusalem artichoke tubers, which are mainly used during processing comprise 75.88% water, 12.46% total sugar, 1.58% total protein (w/w), 1.77% total fat, and other microelements as well as vitamins [17]. Among them, inulin accounted for 80% of the carbohydrates found in tubers (Table 1) [14, 16]. Generally, differences among cultivars, harvest periods, production conditions, postharvest storage, and processing methods result in variations. As a main component of tubers, inulin is a storage polysaccharide found in many plants. The inulin mostly consists of d-fructose bonded by (2→1) β-linkages that are terminated by d-glucose molecules bonded to fructose by (2→1) α-bonds. The degree of polymerization (DP) of standard inulin ranges from 2 to 60 and the average molecular weight is approximately 5500 Da [18]. Besides the presence of abundant fermentable polysaccharides, the Jerusalem artichoke is rich in amino acids, B vitamins, and a variety of minerals that are advantageous for multiproduct biorefineries.

**Table 1:** Chemical composition of Jerusalem artichoke. Data from Kaldy et al. [17]

Ingredient	Content (% of fresh weight)
Water	75.88
Dry mass	24.12
Total sugar	12.46
Total protein	1.58
Total fat	1.77
P, K, Ca, Mg, Fe	0.50
Vitamin A (I.U. in fresh weight)	37
Vitamin C (mg/100 g fresh weight)	0.82

### Jerusalem artichoke pretreatment

An efficient inulin extraction process is important to ensure the successful utilization of Jerusalem artichoke in many fields. Fresh tubers are washed, dried, and ground into a crude powder using a crushing machine over a 40-mesh screen. Due to the high solubility of inulin in hot water, extracting inulin from the powder only requires a straightforward hot water extraction technique. Wei et al. optimized the conditions by using a neutral pH for 20 min at 76.65 °C and solvent:solid ratios of 10.56:1 (v/w); an inulin extraction yield of 83.6% was obtained [19]. Yi et al. extracted inulin from fresh, frozen, or dry tubers through hot water extraction, and showed that the highest yield of 93% was obtained from Jerusalem artichoke powder [20]. When Jerusalem artichoke is used in industrial biorefinery, the inulin extract is firstly degraded into fermentable sugars for microbial utilization through acid or enzyme hydrolysis. Acid hydrolysis is an early method for inulin hydrolysis. The acid hydrolysis of inulin has been investigated utilizing mainly sulfuric or hydrochloric acid. Nasab et al. investigated the effects of pH, temperature, and time on acid hydrolysis of inulin and the maximum amount of inulin hydrolysis (> 90%) was obtained at the pH < 2, temperature > 90 °C and the time of 1 h [21]. Xu et al. obtained 92.3% of the total sugars (20.2 ± 0.1 g/L of glucose and 87.5 ± 0.3 g/L of fructose) from 120 g/L inulin hydrolyze with 5% (w/w) HCl at 60 °C for 1 h [22]. Therefore, acid hydrolysis of inulin has lower cost and proceeds faster. However, acid hydrolysis results in colored by-products and inhibitors that interfere with microbial growth [23]. And nowadays, more and more attentions are paid to the development of green production technology, and acid hydrolysis is not fit with this idea. So, researchers begin to focus on the enzyme hydrolysis. Inulinase is the most commonly used for inulin hydrolysis. Sarchami et al. optimized the enzymatic hydrolysis of inulin from Jerusalem artichoke tubers and the 94.5% of the inulin conversion was achieved at 48 °C for 48 h with the inulinase loading of 10 units g/inulin [24]. Singh et al. used immobilized yeast inulinase for the hydrolysis of inulin in a batch system and maximum hydrolysis of inulin 84.5% was observed at 125 rpm after 4 h [25]. Szambelan et al. compared the acid and enzymatic hydrolysis in Jerusalem artichoke tubers pretreatment and showed that acid and inulinase hydrolysis produced 178.8 and 68.8 g reducing sugars per kg wet matter of tuber for 1 h, respectively [26]. Compared with acid hydrolysis, enzymatic hydrolysis has mild reaction, less by-products, no pigmentation, and is easier to separate and refine. Furthermore, the unique advantage of enzymatic method is that it can simultaneously combine hydrolysis and fermentation in Jerusalem artichoke biorefinery, which shorten the overall time and improve the productivity [27]. In addition, some microorganisms can directly produce inulinases to hydrolyze inulin and avoid the addition of enzymes during refining, which greatly reduces the cost [28, 29]. With the popularity of green production technology, the use of enzymatic degradation of inulin has become a major trend in the industry. After hydrolysis, the composition of Jerusalem artichoke hydrolysate was determined using the method previously described by Kaldy et al. [17].

## Microbial inulinases for biorefinery

During the process of Jerusalem artichoke biorefining, degradation of inulin into fermentable sugars for microbial utilization is the key step. Inulin is mainly broken down through acid hydrolysis and enzymatic hydrolysis into fermentable sugars for biorefinery. Compared with acid hydrolysis of inulin, inulinase hydrolysis has the advantages of less by-products, no pigmentation, and is easier to separate and refine, which is more suitable for fermentation production. As a type of furan fructose hydrolase, inulinase can act on the β-2,1 glycosidic bond, converting inulin into glucose, fructose, or inulooligosaccharides [30]. The main commercial source of inulinase is microorganisms due to the ease of large-scale cultivation and their high inulin hydrolysis activity. At present, inulinase-producing microorganisms can be screened using a plate assay as previously described by Castro et al. [31]. Table 2 lists the high inulinase activity of different inulinase-producing microorganisms. To isolate inulinase overproducers, a number of inulinase-producing strains have been reported. Kango et al. isolated a new strain, *Aspergillus niger* NK-126, and used dandelion tap root extract as the medium substrate for inulinase production; inulinase activity reached a final concentration of 55.0 U/mL after 96 h of fermentation [32]. Ge et al. obtained the mutant strain *A. niger* SL-09 by using UV light and LiCl, whose inulinase 160 activity increased nearly twofold (150 U/mL). For the *A. niger* SL-09 strain, the culture medium was further optimized by using sucrose ester that can act as an effective inducer of inulinase synthesis; the highest inulinase activity reached 230 U/mL after 96 h of shake flask fermentation at 30 °C with 140 rpm [33]. Moreover, yeast strains were also proven as another predominant inulinase producer. *Kluyveromyces marxianus* is the most common inulinase producer that has been studied extensively. A recently isolated strain, *K. marxianus* YS-1, was researched for its inulinase production [34]. After condition optimization, maximum inulinase activity reached 55.4 U/mL after 60 h at an agitation rate of 200 rpm and aeration of 0.75 vvm when using 2% of the inulin extracted from dahlia tubers. *K. marxianus* ATCC 16045 was also reported to produce a large amount of inulinase (121 U/mL) and was proposed as a future inulinase overproducer in yeasts [35]. Additionally, some marine yeast were also found to efficiently synthesize inulinase. After screening over 300 marine yeast strains from different marine environments, *Cryptococcus aureus* G7a, a yeast found in sediments from the South China Sea, was found to produce the largest amount of extracellular inulinase. Under optimal conditions, over 85.0 U/mL of inulinase activity was produced within 42 h of shake flask fermentation [36]. Fang et al. selected the yeast strain, *Pichia guilliermondii*, from marine algae. Using optimal culture conditions, inulinase activity reached 61.5 ± 0.4 U/mL after 48 h [37]. To further increase inulinase activity of the strain, mutant strain M-30, with a high inulinase activity (115.0 ± 1.1 U/mL), was obtained after mutagenesis by UV and LiCl. When using response surface methodology (RSM) to optimize composition and conditions of the medium, inulinase activity of the M-30 strain ultimately reached 127.7 ± 0.6 U/mL [38]. Bacteria are important microorganisms in biorefinery and the inulinases they produce are stable at a wide range of pH values and higher temperatures, indicating application value. A thermophilic inulinase-producing strain was isolated from different soil samples using inulin as the sole carbon source and culturing at 50 °C; fermentation conditions of *Bacillus smithii* T7 were optimized, and inulinase activity increased to 135.2 U/mL after incubating for 72 h [39]. This is the highest reported inulinase activity produced by bacteria thus far.

**Table 2:** Different inulinase-producing microorganisms and inulinase activity

Source	Type	Strains	Enzyme activity	Enzyme property						References
				Mr (kDa)	Optima		Kinetic characteristics		Effects of metal ions	
					T (°C)	pH	*K*_m_	*V*_m_		
Molds	Exo-	*Aspergillus fumigatus*		62	60	6.0	1.25 mM	3.47 × 10^4^/min	Activated by K^+^ and Cu^2+^; inhibited 5 mM Hg^2+^ and Fe^2+^	[49]
	Exo- Endo-	*Aspergillus niger* NK-126	52.3 U/mL	65	55	5.0	5.3 ± 1.1 mM	402.1 ± 53.1 µmol/min/mg	Activated by Cu^2+^; inhibited by Fe^2+^	[32, 44]
	Exo-	*Aspergillus niger* SL-09	230 U/mL							[33]
	Exo-	*Aspergillus niveus* 4128URM	11 U/mL		45	4.0 and 4.8				[61]
	Exo-	*Aspergillus ficuum* SK004	53.1 U/mL		60	4.5				[62]
	Exo- Endo-	*Aspergillus ficuum* JNSP5-06	205.6302 U/gds	Exo-I: 70 Exo-II: 40 Exo-III: 46 Endo-I: 34 Endo-II: 31	45	Exo-I: 4.5 Exo-II: 4.5 Exo-III: 4.5 Endo-I: 5.0 Exo-II: 5.0	Exo-I: 43.1 mg/mL Exo-II: 31.5 mg/mL Exo-III: 25.3 mg/mL Endo-I: 14.8 mg/mL Endo-II: 25.6 mg/mL		Inhibited by Ag^+^ Fe^2+^ and Al^3+^	[63, 64]
	Endo-	*Aspergillus tritici*	25.01 U/mL							[65]
	Endo-	*Penicillium* sp. TN-88	9.9 U/mL	68	50	5.2	0.20 mM at 40 °C and pH 5.0		Inhibited by Ag^+^ and Hg^+^	[54]
Yeasts	Exo-	*Kluyveromyces marxianus* YS-1	55.4 U/mL		50	5.5			Activated by Mn^2+^ and Ca^2+^; inhibited by Hg^2+^ and Ag^2+^	[34, 47]
	Exo-	*Kluyveromyces* sp. S120	409.8 U/gds		50	5.5	10.63 mg/mL	15.72 mg/(mL s)	Activated by Mn^2+^, Mg^2+^ and Ca^2+^; inhibited by Zn^2+^, Cu^2+^ and Fe^2+^	[66, 67]
	Exo-	*K. marxianus* ATCC 16045	121 U/mL	59						[68]
	Exo-	*Cryptococcus aureus* G7a	85.0 ± 1.1 U/mL	60.0	50	5	20.0602 mg/mL	0.008502 mg/min	Activated by Ca^2+^, K^+^, Na^+^, Fe2 + and Cu2 + ; inhibited by Mg^2+^, Hg^2+^, and Ag^+^	[69, 70]
	Exo-	*P. guilliermondii* M-30	127.7 U/mL	57.6	60	6				[38, 71]
Bacteria	Exo-	*Streptomyces* sp. GNDU-1	0.552 U/mL		60	5.5				[72]
	Exo-	*Paenibacillus polymyxa* ZJ-9	0.45 U/mL	56	25	6.0	1.72 mM	21.69 μmol/min/mg	Activated by Zn^2+^, Fe^2+^, and Mg^2+^; inhibited by Co^2+^, Cu^2+^	[73]
	Endo-	*Arthrobacter* sp. S37	10.8 U/mL	75	50	7.5				[74, 75]
	Endo-	*Bacillus smithii* T7	135.2 U/mL		70	4.5				[39]
	Endo-	*Bacillus safensis* AS-08	28.67 U/mL							[76]

**Fig. 2:**
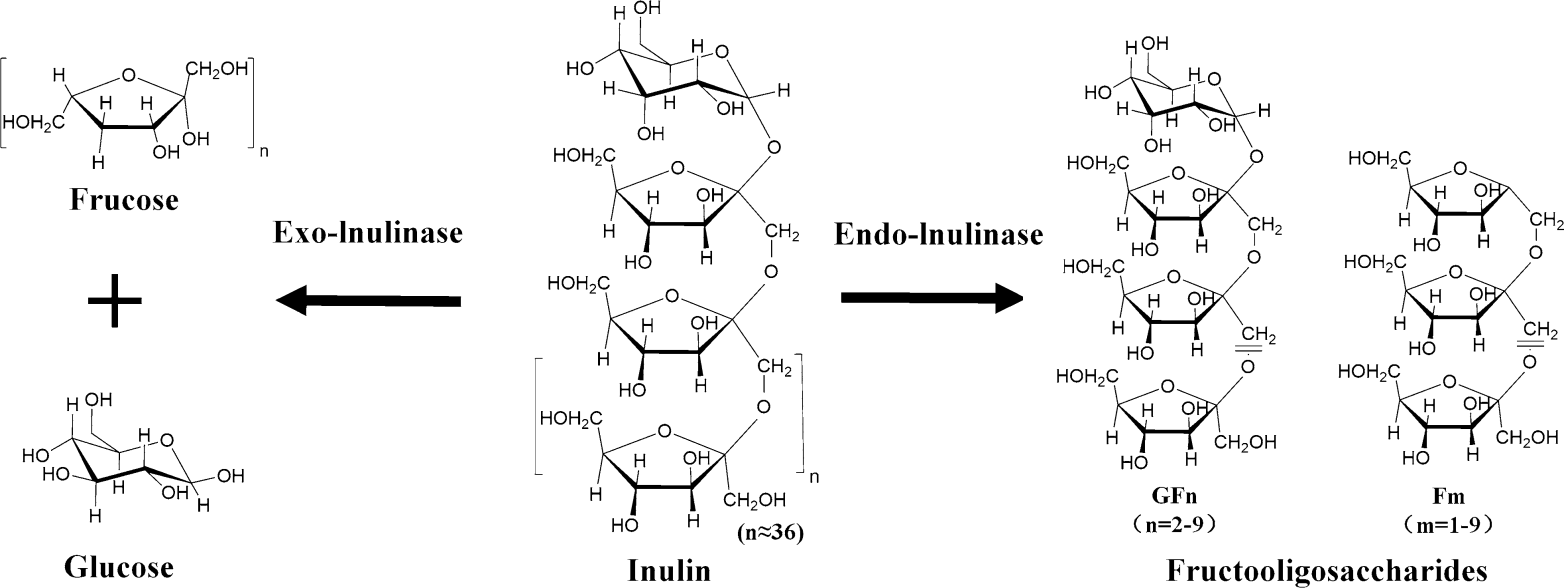
Two modes of action on inulin by inulinases

Based on the differential hydrolysis of glycosidic bonds, inulinase can be divided into exo-inulinases (E.C. 3.8.1.80) or endo-inulinases (E.C. 3.2.1.7). Exo-inulinase functions by removing individual fructose from the non-reducing end of inulin. Endo-inulinase can randomly break the β-2,1 glycosidic bonds in inulin, and can be used to produce inulotrioses (F3), inulotetraoses (F4), and other IOSs that can be designated as GFn or Fn, where G and F stand for glucose and fructose, respectively (Fig. 2) [40]. The classical method of inulinase activity determination is the Nelson–Somogyi colorimetric method [41].

### Catalytic properties and mechanism of exo-inulinase

The exo-inulinase gene, *INUI*, was firstly cloned by Laloux et al. from *K. marxianus* ATCC 12424, and it had an ORF of 1668 bp and 555 encoded amino acids [42]. Then Wen et al. reported on the gene encoding exo-inulinase from *K. cicerisporus* CBS4857, and found that the gene structure contains an ORF of 1665 bp that encodes 555 amino acids, including a signal peptide composed of 23 amino acids [43]. The properties of inulinases from different microorganisms are summarized in Table 2. The molecular weight of exo-inulinase ranges from 40.0 to 256.0 kDa. As reported, most of the exo-inulinases derived from fungi have a greater molecular weight than 50.0 kDa, such as *C. aureus* G7a (60.0 kDa), *P. guilliermondii* strain1 (50.0 kDa), and *A. niger* 12 (65.0 kDa) [36, 37, 44]. The optimal pH ranges were 5.0–6.5 for yeast exo-inulinase, 4.0–6.0 for mold exo-inulinase, and 4.5–7.5 for bacterial exo-inulinase. For industry application and increasing solubility of inulin, thermal stability would be an advantageous property of inulinase. The optimal temperature of inulinase produced by yeast is usually 50 °C [45–47], while it was 60 °C for exo-inulinases from *Aspergillus ficuum* JNSP5-06 [48] and *Aspergillus. fumigatus* [49]. In addition, the optimal temperature for exo-inulinases produced from *B. polymyxa* MGL21 is 35 °C [50]. The results indicate that optimal temperatures of inulinases produced by various microorganisms are different.

**Fig. 3:**
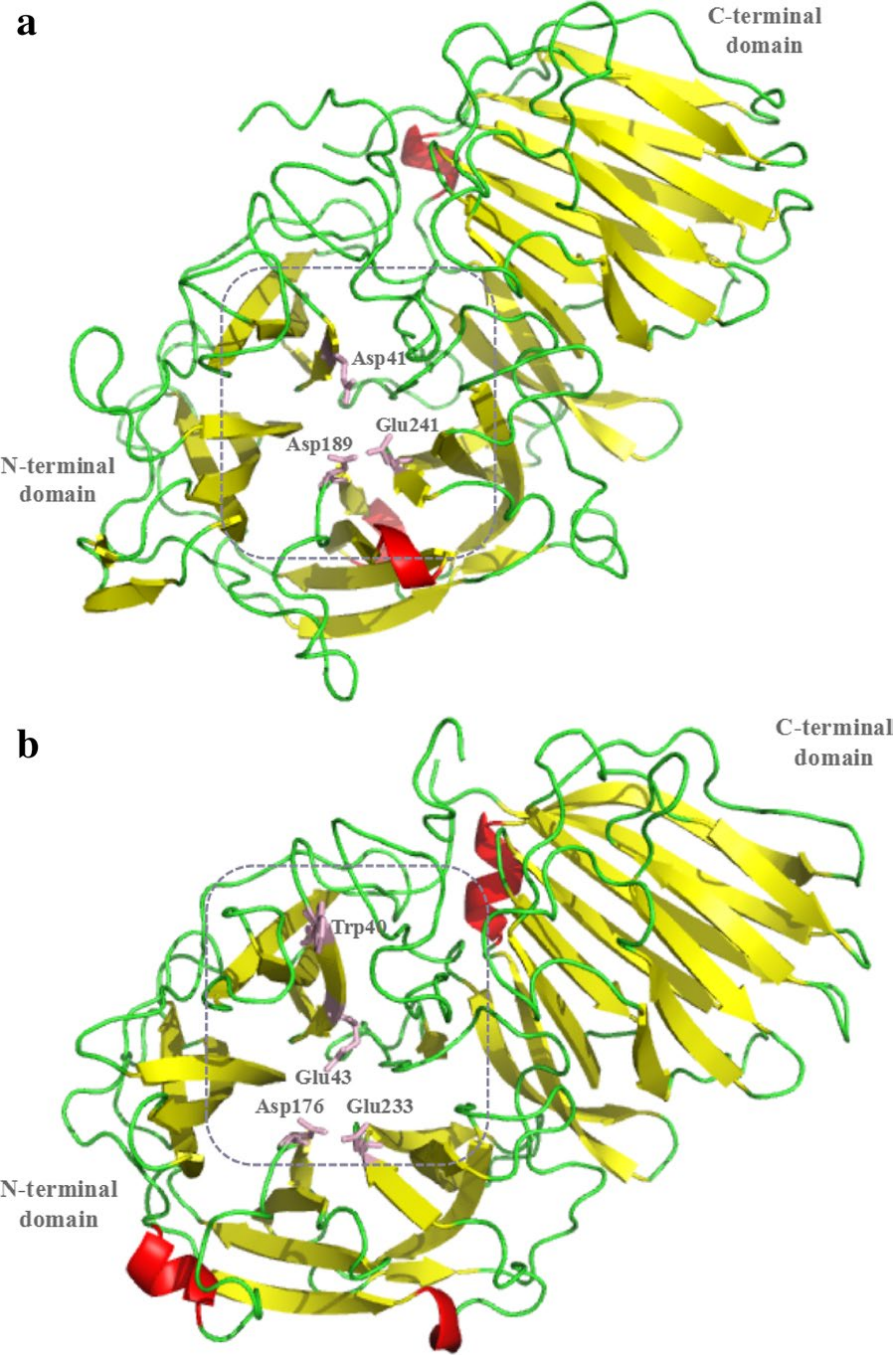
**a** Stereoview of the catalytic site of exo-inulinase from *Aspergillus awamori*. **b** Structural rearrangements in the EnIA putative catalytic site of endo-inulinase from *Arthrobacter* sp. S37

At present, most of works on inulinase are focused on screening of inulinase overproducers and extraction and purification of the enzyme. There are only a handful of studies on the mechanisms of inulinase. The crystal knot of exo-inulinase derived from *Aspergillus awamori* was determined for the first time by X-ray diffraction by Nagem et al. [51]. As a member of the G32 family of glycosyl hydrolases, exo-inulinase consists of two domains, an N-terminal comprising 353 amino acid residues (Phe20 to Gln372) and a C-terminal with 156 amino acid residues (Arg382 to Asn537). The N-terminal domain is the catalytic region of exo-inulinases and is composed of 5 leaf-like helical fold structures. In the conserved sequence 38-WMNDPNG-44 [motif A], the Asp41 residue acts as a nucleophile to attack the substrate, forming the intermediate between enzyme and substrate, and in the sequence 241-ECPGL-245 [motif F], Glu241 functions as a general acid/base catalyst that constitutes the active site of the enzyme (Fig. 3a). Arg188 and Arg189 of the E motif (RDPKV) participate in the recognition and binding of substrates. Moreover, Goosen et al. studied the C-terminal conserved G motif (SVEVF) of *A. niger* exo-inulinases and found that when Ser469 was replaced with threonine residues, hydrolysis activity of the mutant in forming sucrose, inulin, and levan, was decreased. When G motif Ser469 was replaced with the hydrophobic valine residue, the enzyme was completely inactivated. The results showed that the G motif is responsible for hydrolysis of fructans and sucrose in the inulinase catalytic process [52].

### Catalytic properties and mechanism of endo-inulinase

**Fig. 4:**
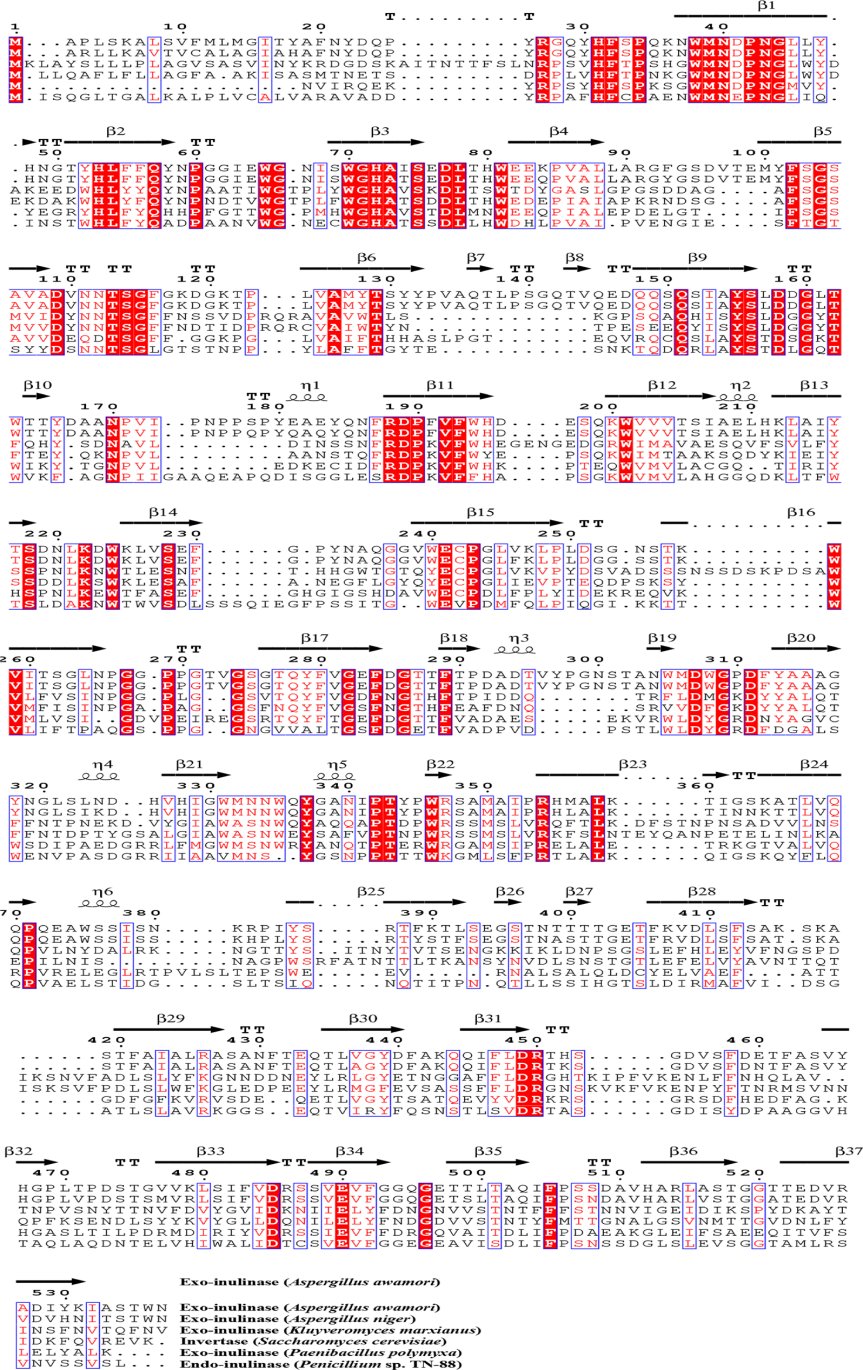
Sequence alignment of inulinase representative members

Due to the health benefits of fructooligosaccharides, the utilization of endo-inulinase to produce fructooligosaccharides from inulin is increasing. An endo-type inulinase containing 555 amino acids was purified from *Penicillium* sp. TN-88 [53]. The enzyme is 68.0 kDa with a high inulinase activity at pH 5.2 and 50 °C. Hydrolysis rates reached 70% after 72 h and the product was mainly composed of F3. Mutanda et al. used the purified endo-inulinase from *A. niger* for fructooligosaccharides production. Molecular mass of the enzyme is 68.1 kDa and can act on inulin substrates at pH 6 and 60 °C, producing high yields of inulotrioses (70.3 mM), inulotetraoses (38.8 mM), and inulopentaoses (3.5 mM) [54]. In addition, bacterial endo-inulinases were also studied; Li et al. reported that the *Arthrobacter* sp. S37 strain can produce endo-inulinase EnIA (2439 bp). The molecular weight is 75.0 kDa, and a pH of 7.5 and a temperature of 50 °C are the optimal conditions for this purified enzyme. The main products of inulin hydrolysis are inulotrioses, inulotetraoses, and inulopentaoses [55]. A novel endo-inulinase-producing strain, *Streptomyces rochei* E87, was identified and found to degrade inulin into inulotriose as the main product with a yield of 71% [56]. In addition, *Xanthomonas* sp. was also implicated in inulin hydrolysis, with the inulooligosaccharide conversion rate of 93% at pH 7, and DP ≥ 5 oligosaccharides [56, 57]. In contrast to exo-inulinases (Fig. 4), the Glu (E) residue in the motif A (WMNEPNG) of endo-inulinases has a key role in the catalytic activity of the enzyme, while the conserved region (SVEVF) is important in anchoring macromolecular glycans and attacking long chain inulin or glycans (Fig. 3b) [58, 59]. Kim et al. studied the functional and catalytic mechanisms of endo-inulinases derived from *Arthrobacter* sp. S37. Three conserved amino acid residues (Glu323, Asp460, and Glu519) from 319-WMNDEPNGL-327 [motif A], 459-RDF-461 [motif E], and 519-ECMP-522 [motif F], respectively, were modified by site-directed mutagenesis. The *k*_cat_ values of E323A and E519A mutants decreased, but there was no variation in *K*_m_, consistent with their putative roles as nucleophiles and acid–base catalysts. On the other hand, the D460A mutant was completely inactive. These results proved that Glu323, Asp460, and Glu519 are essential amino acids for enzyme activity [60].

## Research advances on the biorefinery potential of Jerusalem artichoke

### Fructooligosaccharides

Fructooligosaccharides have been classified as prebiotics due to their bifidogenic nature and health-promoting properties when consumed in sufficient amounts as recommended by health practitioners [77, 78]. From an industrial point of view, FOSs are mainly enzymatically manufactured from sucrose using fructosyltransferases (DP < 4) or by controlled enzymatic hydrolysis of inulin (DP < 9) [79]; however, the former process is complex as several reactions occur and is inevitably accompanied by a residual of sucrose, that can lead to food-related obesity [80]. By contrast, the production of FOSs from inulin using endo-inulinases is a single-step process. Moreover, FOSs produced from inulin contain longer chains (DP = 2–9) that have enhanced physiological activity than those synthesized from sucrose (DP = 2–4). Recent advances in industrial enzymology have made the large-scale production of FOSs from inulin possible.

It has been reported that many filamentous fungi and bacterial strains can produce endo-inulinases [81]. Endo-inulinase from *pseudomonas* sp. was used for FOSs (DP = 2–7) production with a yield of 75.6% [82]. At the same time, Yun et al. reported FOSs yields of 83% from inulin by using immobilized endo-inulinase from *Pseudomonas* sp. The immobilized endo-inulinase reactor operated for 15 days for FOSs production without a significant loss in enzyme activity which indicated the potential of industrial application [83]. Batch-wise production of FOSs from pure inulin was performed using partially purified endo-inulinases from *Xanthomonas* sp. obtaining a yield of 86% and DP = 5–6 of the main product [57]. Cho et al. developed a dual endo-inulinase system from *Xanthomonas* sp. and *Pseudomonas* sp. for the maximum FOSs yields of 92% from pure inulin. In addition to FOSs production from inulin by bacterial endo-inulinases, molds are also important sources of endo-inulinase [84]. Jin et al. purified endo-inulinase from *Aspergillus ficuum* and achieved FOSs (DP = 2–8) production of 80% using the Jerusalem artichoke juice as substrate after 72 h [85]. A newly discovered producer *Aspergillus trtici* BGPUP6 with 25.01 U/mL of endo-inulinase was considered as a candidate for FOSs production [65]. Due to the low endo-inulinase activity by native microorganisms, recombinant endo-inulinases have been also used to produce FOSs from inulin in recent years. Yun et al. first cloned the endo-inulinase gene, *INU1*, from *Pseudomonas* sp. and expressed constitutively in *Escherichia coli* HB101 for FOSs production [86]. A yield of 78% was obtained by using immobilized cells at 50 °C and continuously operated for 17 days without endo-inulinase loss. *E. coli* BL21 (DE3) was also used to express the endo-inulinase isolated from *Aspergillus ficuum*; the highest endo-inulinase enzyme activity (75.22 U/mg) was obtained and an IOS yield of 94.41% was achieved at 55 °C and pH 4.6 for 24 h [87]. Although a high FOSs conversion rate was achieved in an *E. coli* expression system, the system’s lack of safety greatly limits its application in the food industry. Kim et al. cloned *INU1* from *Pseudomonas mucidolens*, after which the expressed endo-inulinases in *Saccharomyces cerevisiae* cells hydrolyzed inulin with an IOS yield of 71.2% after 30 h; the IOSs mainly consisted of inulotetraoses [88]. On the other hand, a high yield of FOS production (91.3%) was obtained from recombinant *Pichia pastoris* expressing the endo-inulinase gene (*EnInu*) from *A. niger* [89]. As an important value-added product from Jerusalem artichoke material, FOSs as function food have attracted extensive attention. By recombinant expressions of endo-inulinase, a handful of reports on the FOSs production with high yields have been presented above which exhibits great potential for industrial applications, and developments in separation of by product (glucose, sucrose) and impurity for high pure FOSs product is the need for future work. To simplify the IOS production process, high-efficiency immobilization technology, novel reactors for biocatalysts should be explored to scale up to industrial production.

### Ethanol

Ethanol is not only an important raw material in the chemical synthesis industry, but it is also the most promising biofuel. Among the biorefinery products of Jerusalem artichoke, ethanol has been studied extensively. However, the most commonly used alcohol production strains in the industry, *S. cerevisiae* and *Zymomonas mobilis*, cannot effectively utilize inulin to produce ethanol, which severely restricts the industrialization of ethanol production of inulin-based biomass [90]. Traditional ethanol production processes firstly convert inulin to fructose via acid hydrolysis, and then reuse the microbes to ferment ethanol. Onsoy et al. used the acid hydrolysates of Jerusalem artichoke juices as the media for ethanol fermentation by *Z. mobilis*, which led to consistent ethanol yields (0.45 g/g) and a conversion efficiency of 83.19% of theoretical value [91]. The cost of inulin acid hydrolysis technology for ethanol production is low, but the process is relatively complicated as hydrolysates often contain HMF, a microbial growth inhibitor, and is not environment friendly [92]. Therefore, researchers begin to use microbes or enzymes to assist with *S. cerevisiae* in producing ethanol directly from inulin. Zhang et al. used the recombinant inulinase produced by *P. pastoris*, *X*-*33/pPICZaA*-*INU1*, to hydrolyze inulin and ethanol fermentation by *S. cerevisiae* sp. W0. The total sugar utilization rate after 120 h of fermentation was 98.9%, and an ethanol yield of 0.384 g/g inulin was obtained [93]. The study further proved that inulinase pretreatment of inulin products for ethanol fermentation is feasible. Although inulinase-assisted *S. cerevisiae* ethanol production is an environmentally friendly technology, the formed fructose inhibits inulinase secretion. Therefore, to relieve product inhibition, researchers have developed a co-fermentation technology on ethanol production combined with inulinase-producing microorganisms. Through mutagenesis, Ge and Zhang obtained an inulinase-producing strain, *A. niger* SL-09, and co-cultured it with the highly ethanol-tolerant *S. cerevisiae* Z-06. After 48 h of Jerusalem artichoke fermentation, the utilization rate of inulin was 98%, and the ethanol concentration reached 19.6% (v/v) [94]. Therefore, there is a need to further optimize microbial strains and the process, by improving microbial fermentation co-culture parameters to maximize efficiency of ethanol production from inulin. Since the genome of *S. cerevisiae* is well studied and genetic manipulation techniques are more advanced, more researchers are focusing on the use of genetic engineering strategies to obtain a recombinant *Saccharomyces cerevisiae* with inulinase that can directly produce ethanol from inulin. Tong et al. expressed the *INU1* gene from marine-derived *P. guilliermondii* and the recombinant *Saccharomyces* sp. W0 was able to produce 34.2 U/mL of extracellular inulinase activity in 72 h. During 2 L fermentation, 14.9% (v/v) of ethanol was obtained with the conversion efficiency of 99.5% from inulin to ethanol [95]. Yuan et al. cloned the exo-inulinase gene from *Candida kutaonensis* and expressed in *S. cerevisiae* for the improvement of inulin utilization, and the recombinant *S. cerevisiae* was able to produce high ethanol yields from both inulin and Jerusalem artichoke tuber flour [96]. A natural engineering *S. cerevisiae* engineered with rational strategies such as co-expressing exo- and endo-inulinase gene, inactivated proteases between haploid and diploid was investigated for inulin utilization to produce ethanol. Ethanol fermentation from 200 g/L inulin and 250 g/L raw Jerusalem artichoke tuber powder resulted in productivity of 2.44 and 3.13 g/L/h, respectively [97]. Actually, in addition to building engineering strains, some yeast genus, such as *Kluyveromyces fragilis* and *Kluyveromyces marxianus* can both produce inulinase and ethanol. Rosa et al. selected *K. marxianus* to utilize extracted juice of Jerusalem artichoke tubers for ethanol production, the production of 12.8% (v/v) of ethanol in 70 h with the consumption of 95% of initial sugars, and an ethanol yield 77% of the theoretical maximum were achieved [98]. Yuan et al. studied ethanol fermentation of *K. marxianus* ATCC8554 using inulin as substrate and the highest ethanol yield of 91.5% of the theoretical value was achieved [99]. Above all, we believe that the use of Jerusalem artichoke in ethanol fermentation will become more economical and practical.

### Biodiesel

Compared with bioethanol, biodiesel has a higher heating value and lower water absorption, and can be used directly in vehicles without engine modification [100]. Cheng et al. determined the feasibility of Jerusalem artichoke tubers as feedstock for biodiesel preparation, expecting to reduce the cost of microalgal cultivation for bio-oil and biodiesel production [101]. In their study, Jerusalem artichoke hydrolysate was used as carbon sources for lipids accumulation by *Chlorella protothecoides*. After 4-day scale cultivation, the lipid concentration of 44% by dry mass was extracted and turned into biodiesel by transesterification. The biodiesel contained oleic acid methyl ester, linoleic acid methyl ester, and cetane acid methyl ester as the main components. Zhao et al. also reported on lipid production from Jerusalem artichoke tubers using the oleaginous yeast, *Rhodosporidium toruloides* Y4. The lipid titer of 39.6 g/L and cellular lipid content of 56.5% (w/w) were obtained when Jerusalem artichoke hydrolysates were fed [102]. Sung et al. used a lab yeast strain of *Cryptococcus* sp. and achieved lipid productivity of 1.73 g/L/d after optimization [103]. These studies suggest the feasibility of an alternative method of producing biodiesel from Jerusalem artichoke tubers using microalgal cultivation, where a cost reduction of carbon source feed in algal oil production can be expected.

### 2,3-Butanediol

2,3-Butanediol (2,3-BD) is an attractive chemical which can be used as a starting material for the manufacture of bulk chemicals such as 1,3-butadiene, methyl ethyl ketone, and so on [104]. As an important platform chemical, the production of 2,3-BD is mainly by chemical or biotechnological methods. With the depletion of crude oil, biotechnological production of 2,3-BD has received more and more attention [105]. Recently, several strains of bacteria and fungi are considered to produce 2,3-BD, including *Aeromonas hydrophila*, *Klebsiella oxytoca*, *Pseudomonas hydrophila*, *Trichoderma harzianum*, *K. pneumoniae,* and *B. polymyxa* [106]. From an economic point of view, Fages et al. were the first to utilize Jerusalem artichoke tubers as the cheap raw material on efficient 2,3-BD production with *B. polymyxa* ATCC 12321. By optimizing *k*_L_*a* profile, 44 g/L of 2,3-BD with a productivity of 0.79 g/L/h was obtained during batch culture [107]. Unfortunately, since then, little attention was paid to the microbial production of 2,3-BD from Jerusalem artichokes. Until 2009, separate hydrolysis and fermentation (SHF) and simultaneous saccharification and fermentation (SSF) were successfully introduced to 2,3-BD production by *K. pneumoniae* from Jerusalem artichoke tubers. In batch SHF process, the high concentration of fructose caused substrate inhibition for growth of cells and target products accumulation, leading to a low 2,3-BD productivity of 1.08 g/L/h while SSF, which was preferable for 2,3-BD production with high concentration (84.03 g/L) and productivity (2.1 g/L h) in fed-batch process [108]. Around the same time, Li et al. also reported that fed-batch SSF using *K. pneumoniae* was successfully performed, and 80.5 g/L of target products (2,3-butanediol and acetoin) were obtained by a stage-shift aeration strategy after 68 h [109]. Li et al. also reported that fed-batch SSF using *K. pneumoniae* was successfully performed, and 80.5 g/L of target products (2,3-butanediol and acetoin) were obtained by a stage-shift aeration strategy after 68 h and used the thermophilic *Bacillus licheniformis* ATCC 14580 for 2,3-BD production from inulin, with the yield of 103.0 g/L in 30 h by fed-batch SSF [105]. This is the highest 2,3-BD production reported by utilizing inulin as the substrate nowadays. However, all the above studies needed separate processes to hydrolyze inulin of Jerusalem artichoke or exogenous inulinase added for 2,3-BD production. On the other hand, some 2,3-BD producers of *Paenibacillus polymyxa* were proven capable of fermenting inulin without previous hydrolysis. Gao et al. developed a one-step fermentation technique of raw inulin extracts from Jerusalem artichoke tubers by *P. polymyxa* ZJ-9 to produce (R,R)-2,3-BD. Under optimal conditions, the concentration of obtained (R,R)-2,3-BD reached 36.92 g/L, at more than 98% optical purity [110]. This process greatly decreased the cost and facilitated its practical application in 2,3-BD production. By genetic engineering technologies, the future work on manufacturing and modifying the industrial strains could have economic benefit on (R,R)-2,3-BD production.

### Lactic acid

As an important platform chemical, lactic acid has been widely used in the many fields such as food, pharmaceutical, and chemical industries. In order to construct a cheap and energy-efficient process for lactic acid production, a variety of work has been undertaken for inexpensive raw substrates. As early as 1942, Andersen and Greaves utilized Jerusalem artichoke tubers to produce d-lactic acid [111]; however, by the 21st century, more researchers have begun to pay attention to the production of l-lactic acid using Jerusalem artichokes. Ge et al. creatively used a mixed-culture fermentation process, where *A. niger* SL-09 and *Lactobacillus* sp. G-02 were used to directly form l-lactic acid from Jerusalem artichoke tubers. The inoculation of l-lactic acid-producing strain *Lactobacillus* sp. G-02 greatly enhanced inulinase and invertase from *A. niger* SL-09, leading to the highest l-lactic acid concentration of 120.5 g/L with a high conversion efficiency of 94.5% after 36 h of fed-batch fermentation [112]. In their study, inulinase activity was subjected to product inhibition in SSF, whereas fermentation activity of *Lactobacillus casei* G-02 was subjected to substrate inhibition. To further enhance lactic acid productivity, Ge et al. supplemented the media with sodium citrate to maximize the specific growth and fructose consumption rates of *L. casei* G-02. As a result, a l-lactic acid yield of 141.5 g/L after 30 h was obtained by supplement of 10 g/L sodium citrate when inoculated a 10% volume of *L. casei* G-02 [113]. Shi et al. provided a way of producing l-lactic acid by immobilized cells. A fibrous-bed bioreactor was used to immobilized *Lactococcus lactis* cells for l-lactic acid production from hydrolysates of Jerusalem artichoke. The maximum l-lactic acid concentration of 142 g/L was obtained in the fed-batch fermentation [114]. Choi et al. found *Lactobacillus paracasei* KCTC13169 could more efficiently ferment Jerusalem artichoke tubers than other *Lactobacillus* spp. 92.5 g/L of lactic acid was achieved by a direct fermentation from Jerusalem artichoke extracts at 111.6 g/L of sugar content without acidic or enzymatic inulin hydrolysis. The conversion efficiency of inulin to lactic acid reached 98% of theoretical yield [115]. Recently, Wang et al. managed to obtain high-optical purity of l-lactate from hydrolysates of Jerusalem artichoke powder by using a thermophilic bacterium, *Bacillus coagulans* XZL4; a concentration of 134 g/L of l-lactic acid was obtained and the optical purity of the final product was 99% [116]. With further research, utilizing Jerusalem artichokes for lactic acid production will become more significant for industrialization.

### Other bio-based products

In addition to the above-mentioned bio-based products, other high value-added intermediate products have been gradually used in the commercial biorefining of Jerusalem artichoke (Table 3). As early as 1985, Marchal et al. investigated acetone–butanol fermentation of Jerusalem artichoke, and used optimized conditions to obtain solvent productions of 23–24 g/L after 36 h [117]. Chen et al. investigated butanol production from acid hydrolysates of Jerusalem artichoke juice by using *Clostridium acetobutylicum* L7; butanol production of this fermentation system reached 11.21 g/L, and the ratio of butanol to acetone to ethanol was 0.64:0.29:0.05 [118]. Sarchami and Rehmann optimized enzymatic hydrolysis of inulin from Jerusalem artichoke tubers for fermentative butanol production, and achieved an acetone–butanol–ethanol yield of 0.33 g/g sugar [119]. Duvnjak et al. were the first to demonstrate the possible use of Jerusalem artichokes for sorbitol production by *S. cerevisiae* ATCC 36859; sorbitol production started after the glucose was entirely consumed from Jerusalem artichoke juice, and when the juice was supplemented with 3% yeast extract, the concentration of sorbitol reached 4.6% [120, 121]. In the study, butyric acid production by immobilized *Clostridium tyrobutyricum* was successfully performed in a fibrous-bed bioreactor (FBB) from Jerusalem artichoke hydrolysates. The high butyric acid concentration of 60.4 g/L was obtained during fed-batch fermentation [122]. Similarly, to enhance propionic acid production from Jerusalem artichoke hydrolysate [123], Gunnarsson et al. investigated the potential of Jerusalem artichoke tuber hydrolysates for succinic acid production using *Actinobacillus succinogenes* 130Z. The concentration of succinic acid reached 52.7 g/L, indicating that Jerusalem artichoke as a feedstock is suitable for succinic acid production by *A. succinogenes*. Jerusalem artichoke is also used in the production of biocompatible and degradable polymer materials [124]. Xia et al. used Jerusalem artichoke hydrolysates for co-production of poly-(l-malic acid) and pullulan by *Aureobasidium pullulans* HA-4D which resulted in poly-(l-malic acid) concentration of 117.502 g/L and pullulan concentration of 15.202 g/L in a 1 t bioreactor [125]. A novel strain *Bacillus amyloliquefaciens* NX-2S was isolated for production of poly-(γ-glutamic acid) by Qiu et al. [28]. The NX-2S strain can assimilate inulin more efficiently than other carbohydrates from Jerusalem artichoke without hydrolytic treatment, and a yield of 39.4 g/L poly-(γ-glutamic acid) was achieved. This was also the first report that utilized Jerusalem artichoke for production of macromolecular compounds.

**Table 3:** Reports on high yields of products biorefined from Jerusalem artichoke

Products	Strain and engineering targets or strategies	Yield	References
Fructooligosaccharides	Endo-inulinase from *Streptomyces rochei*	70%	[56]
	Endo-inulinase from *Pseudomonas* sp.	75.6%	[82]
	Soluble and immobilized endo-inulinase from *Pseudomonas* sp.	83%	[83]
	Endo-inulinase from *Xanthomonas* sp.	86%	[57]
	A dual endo-inulinase system originated from *Xanthomonas* sp. and *Pseudomona*s sp.	92%	[84]
	Endo-inulinase from *Aspergillus ficuum* expressed in *Escherichia coli* BL21 (DE3)	94.41%	[87]
	The displayed endo-inulinase from *Pseudomonas mucidolens* on the cells of *Saccharomyces cerevisiae*	71.2%	[88]
	Endo-inulinase from *Aspergillus niger* expressed in *Pichia pastoris*	91.3%	[89]
Ethanol	*Saccharomyces cerevisiae* KCCM50549	36.2 g/L	[29]
	*Saccharomyces cerevisiae* Bc16a mixed with *Kluyveromyces fragilis* ŁOCK 0027	74.2 g/L	[126]
	*Zymomonas mobilis* 3881	78.1 g/L	[127]
	*Zymomonas mobilis* TISTR 548	79.8 g/L	[91]
	*Saccharomyces cerevisiae* MK01 engineered with inulinase expression through cell surface display	89.3 g/L	[128]
	*Kluyveromyces marxianus* ATCC8554	93.4 g/L	[99]
	An Engineered *Saccharomyces cerevisiae* JZH	95.19 g/L	[97]
	*Saccharomyces* sp. W0	95.5 g/L	[95]
	*Zymomonas mobilis* TRSTR548	95.9 g/L	[129]
	*Kluyveromyces cicerisporus*	96.3 g/L	[130]
	*Saccharomyces cerevisiae* DTN	109.4 g/L	[131]
	*Saccharomyces cerevisiae* Z-06 mixed with *Aspergillus niger* SL-09	154.7 g/L	[94]
Biodiesel	*Chlorella protothecoides* using hydrolysate of Jerusalem artichoke	7.1 g/L	[100]
	*Rhodotorula mucilaginosa* TJY15a	10.2 g/L	[132]
	*Rhodosporidium toruloides* Y4	39.6 g/L	[102]
2,3-Butanediol	*Paenibacillus polymyxa* ZJ-9	36.92 g/L	[110]
	*Bacillus polymyxa* ATCC 12321	44 g/L	[107]
	*Klebsiella pneumoniae*	91.63 g/L	[108]
	*Bacillus licheniformis* ATCC 14580	103.0 g/L	[105]
	A recombinant *Bacillus* sp. strain BRC1 with increased inulinase activity	28.6 g/L	[133]
Lactic acid	*Lactobacillus paracasei* KCTC 13169	92.5 g/L	[115]
	*Aspergillus niger* SL-09 and *Lactobacillus sp*. G-02	120.5 g/L	[112]
	*Bacillus coagulans* XZL4	134 g/L	[116]
	*Lactobacillus casei* G-02	141.5 g/L	[113]
	Immobilized *Lactococcus lactis* cells in a fibrous-bed bioreactor system	142 g/L	[114]
Acetone–butanol	*Clostridium saccharobutylicum* DSM 13864	9.6 g/L	[119]
	*Clostridium acetobutylicum*	23.0–24.0 g/L	[117]
	*Clostridium acetobutylicum* L7	11.21 g/L	[118]
Sorbitol	*Zymomonas mobilis* ATCC 31821	26 g/L	[121]
	*Saccharomyces cerevisiae* ATCC 36859	46 g/L	[120]
Butyric acid	*Clostridium tyrobutyricum Z*JU 8235	60.4 g/L	[122]
Propionic acid	*Propionibacterium acidipropionici* ATCC 4875	26.2 g/L	[123]
Succinic acid	*Actinobacillus succinogenes* 130Z	52.7 g/L	[124]
Poly-(l-malic acid)	*Aureobasidium pullulans* HA-4D	117.502 g/L	[125]
Poly-(γ-glutamic acid)	*Bacillus amyloliquefaciens* NX-2S	39.4 g/L	[28]

## Conclusions and future prospects

With the rapid development of synthetic biology, more high value-added natural products and chemicals are being designed and synthesized by modular processing of metabolic pathways, and assembly and optimization of chassis. In the future, multi-products will be produced by synthetic biological processes, which will lead to huge consumptions of carbohydrate resources. Undoubtedly, using Jerusalem artichoke as a representative non-grain raw material would be an excellent place to start. It is necessary to construct a cell factory for the efficient use of inulin, to be further used as a highly efficient refining platform for the production of a variety of biologically important natural products and synthetic compounds.

For non-food raw materials, the Jerusalem artichoke has unique advantages, such as salt and drought tolerance, and strong adaptability to soil—especially for cultivation in saline–alkaline soils or coastal shoals for ecological environment protection. Meanwhile, as a promising feedstock for bioproduct synthesis, the inulin-containing crop has a high potential for use in the inulin extraction process. Although recent research on Jerusalem artichoke biorefinery is presented in this review, there is less industrial coverage. Key obstacles are how to improve inulinase activity and reduce cost, which remain the major limiting factors of the process. In high-throughput technology and protein engineering, high-activity inulinase resources are still underexploited when it comes to improving biorefinery efficiency. Furthermore, extensive research can be conducted to enhance the fermentation process through a variety of approaches, such as optimization of fermentation parameters, inulinase immobilization technologies, and advanced bioreactor designs for improving the efficiency of Jerusalem artichoke refinery products. This article reviewed the research progress on various Jerusalem artichoke components and discussed their feasibility for future biorefining of Jerusalem artichoke. We hope that this review will contribute to the industrialization of Jerusalem artichoke biorefinery.

## Abbreviations

DP: degree of polymerization
FOSs: fructooligosaccharides
2,3-BD: 2,3-Butanediol
SHF: separate hydrolysis and fermentation
SSF: simultaneous saccharification and fermentation
